# Identifying the Main Functional Pathways Associated with Cognitive Resilience to Alzheimer’s Disease

**DOI:** 10.3390/ijms22179120

**Published:** 2021-08-24

**Authors:** Marta Pérez-González, Sara Badesso, Elena Lorenzo, Elizabeth Guruceaga, Alberto Pérez-Mediavilla, Ana García-Osta, Mar Cuadrado-Tejedor

**Affiliations:** 1 Alzheimer’s Disease, Neurosciences Program, Center for Applied Medical Research (CIMA), University of Navarra, 31008 Pamplona, Spain; m.gonzalez@ucl.ac.uk (M.P.-G.); sbadesso@alumni.unav.es (S.B.); elr@unav.es (E.L.); lamediav@unav.es (A.P.-M.); 2 IdiSNA (Navarra Institute for Health Research), 31008 Pamplona, Spain; eguruce@unav.es; 3 Department of Pathology, Anatomy and Physiology, School of Medicine, University of Navarra, 31008 Pamplona, Spain; 4 Bioinformatics Platform, Centro de Investigación Médica Aplicada, Instituto de Investigación Sanitaria de Navarra, ProteoRed-ISCIII, University of Navarra, 31008 Pamplona, Spain; 5 Department of Biochemistry and Genetics, School of Sciences, University of Navarra, 31008 Pamplona, Spain

**Keywords:** Alzheimer’s disease, cognitive resilience, inflammation, CD4^+^ cells

## Abstract

Understanding the mechanisms involved in cognitive resilience in Alzheimer’s disease (AD) represents a promising strategy to identify novel treatments for dementia in AD. Previous findings from our group revealed that the study of aged-Tg2576 cognitive resilient individuals is a suitable tool for this purpose. In the present study, we performed a transcriptomic analysis using the prefrontal cortex of demented and resilient Tg2576 transgenic AD mice. We have been able to hypothesize that pathways involved in inflammation, amyloid degradation, memory function, and neurotransmission may be playing a role on cognitive resilience in AD. Intriguingly, the results obtained in this study are suggestive of a reduction of the influx of peripheral immune cells into the brain on cognitive resilient subjects. Indeed, *CD4* mRNA expression is significantly reduced on Tg2576 mice with cognitive resilience. For further validation of this result, we analyzed *CD4* expression in human AD samples, including temporal cortex and peripheral blood mononuclear cells (PBMC). Interestingly, we have found a negative correlation between *CD4* mRNA levels in the periphery and the score in the Mini-Mental State Exam of AD patients. These findings highlight the importance of understanding the role of the immune system on the development of neurodegenerative diseases and points out to the infiltration of CD4^+^ cells in the brain as a key player of cognitive dysfunction in AD.

## 1. Introduction

Alzheimer’s disease (AD), which is the most common form of dementia in the elderly, is characterized by the presence in the brain of extracellular deposition of amyloid-β (Aβ) in the form of plaques and intracellular accumulation of hyper-phosphorylated Tau (p-Tau) protein as neurofibrillary tangles. Currently, there is not an effective treatment for AD and the clinically approved drugs have demonstrated limited success in altering AD progression [1]. Indeed, although aducanumab (monoclonal antibody against Aβ) has recently been conditionally approved by FDA, its efficacy in slowing memory loss has yet to be demonstrated. Like many other drugs focused on clearing pathological β-amyloid, this new therapy has revealed a very modest effect on slowing disease progression [2]. These drug failures combined with the socioeconomic burden associated with the disease, highlight the urgent need of finding alternative approaches to the search of new effective AD treatments.

In line with this idea, a very exciting fact is that several clinical studies have shown that some elderly people accumulate a significant number of histopathological AD lesions in their brains despite the absence of signs of dementia [3,4]. Understanding the mechanisms underlying this cognitive resilience appears to be a promising line of research. However, until now, most of the studies showing discordances between cognition and AD-neuropathology are descriptive and only compare the pathological phenotype of demented patients vs. non-demented individuals and their life style. Indeed, most of them point out to a combination of some genetic and environmental factors as causes of the resilience [5,6]. It is intriguing that, similar to humans, about 20% of transgenic mice of AD with substantial AD pathology do not develop memory deficits [7,8]. Taking this into account and considering that there has been little investigation of the impact of neurobiological and genetic factors on cognitive resilience, animal models of AD may represent suitable tools to identify the genes involved in cognitive resilience.

By using this approach, a previous study using transcriptomic analysis (Affymetrix arrays) in the hippocampus of AD mice with preserved memory compared to memory impaired mice, allowed us to identify differentially expressed genes involved in memory preservation [8]. Specifically, the identification of the cytosolic phospholipase PLA2G4E and its target validation lead us to point toward this enzyme as a novel therapeutic target to treat cognitive dysfunction [8]. Similarly, by using proteomics on the 5xFAD mouse model of AD and on control mice with normal or impaired cognition, Neuner et al. have highlighted the importance of HDAC4 as a global regulator of memory [7]. Recently, the same group characterized individual differences in the cognitive abilities during adulthood and investigated evidence of cognitive reserve and/or resilience in middle-aged B6-BXDs mice. By employing RNASeq, they found that gene networks related to transcription/translation, cellular metabolism and neuronal function were associated with working memory, contextual fear memory and cognitive decline [9].

Here, in order to further elucidate mechanisms involved in cognitive resilience, we carefully selected resilient aged-Tg2576 mice by using the Morris Water Maze (MWM) test, as previously described by Pérez-González et al., 2020 [8] and compared them to their counterpart-impaired littermates. In this case, taking into account that Tg2576 mice represent an amyloidosis model of AD and that amyloid plaques are more abundant within cortical brain areas [10], the prefrontal cortex (PFC) was the selected study region. In addition to the hippocampus, PFC is one of the most affected areas in AD [11], accounting for executive dysfunction and other cognitive deficits observed in AD patients [12]. A RNASeq followed by Gene Ontology (GO) and Ingenuity Pathways Analysis (IPA) was performed in the prefrontal cortex to identify novel pathways and/or genes involved in cognitive resilience in AD. These studies unveiled the participation of several pathways involved in synaptic transmission and memory function, but also in the activation of the immune system on the resistance to dementia, pointing out to CD4 infiltration as a direct mediator of cognitive impairment in AD.

## 2. Results

### 2.1. Identification of Cognitive Resilient Tg2576 Mice

**Figure 1:**
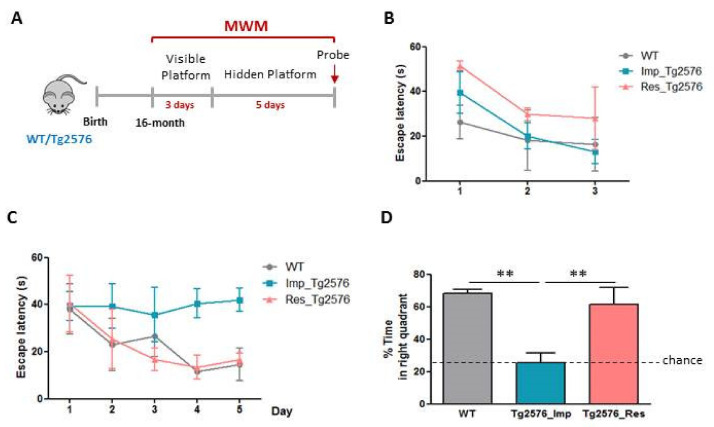
Identification of Tg2576 cognitive resilient mice through MWM test. (**A**) Diagram showing the MWM paradigm used to identify aged-Tg2576 mice with cognitive resilience. (**B**) Escape latency to the visible-platform phase of the MWM test for WT, Imp_Tg2576 and Res_Tg2576 mice. (**C**) Escape latency to the hidden-platform in the MWM test for WT, Imp_Tg2576 and Res_Tg2576 mice. (**D**) Percentage of time spent in the correct quadrant during the probe trial on day 6 (one way ANOVA test followed by Newman-Kewls post hoc test, *n* = 3–4, ** *p* ≤ 0.01).

As we have previously described, the MWM paradigm is a useful method to identify cognitive-resilient Tg2576 individuals [8]. Here, we carried out this behavioral task with 16 months-old wild-type (WT) and Tg2576 animals (Figure 1A) and, as expected, we were able to subdivide Tg2576 mice into cognitive resilient (Res_Tg2576) and impaired (Imp_Tg2576) according to their accomplishment in the test. As depicted in Figure 1B, performance in the visible platform phase of the test showed no-differences between the three groups, indicating that all of them were able to efficiently execute the task (two way ANOVA: effect of day F(2,24) = 3.835, *p* = 0.0359; effect of group F(2,24) = 2.372, *p* = 0.1148; day × group interaction F(4,24) = 0.2606, *p* = 0.9002). Latency times to reach the platform during the hidden-platform phase of the test were similar between WT and resilient individuals and lower than those of demented mice, indicating that, unlike Imp_Tg2576 mice, Res_Tg2576 mice did not present spatial memory impairments (two way ANOVA: effect of day F(4,40) = 1.906, *p* = 0.1282; effect of genotype F(2,40) = 6.481, *p* = 0.0036; day × genotype interaction F(8,40) = 0.6916, *p* = 0.6965). In the same line, memory retention was not affected in Tg2576 individuals with cognitive resilience, as the percentage of time that they swam in the correct quadrant of the pool during the probe trial was similar to that of WT mice and significantly higher than the one of impaired mice (one way ANOVA with group as variable: F(2,8) = 14.42, *p* = 0.0022).

### 2.2. Most Differentially Expressed Genes between Resilient and Impaired Aged-Tg2576 Mice Are Involved in Inflammation, Amyloid Degradation, Memory Function, and Neurotransmission

RNA sequence analysis followed by Gene Ontology (GO) and Ingenuity Pathways Analysis (IPA) was performed in the prefrontal cortex to assess the differential gene expression profile between resilient and impaired aged-Tg2576 mice. This area, which is implicated in motor behaviours, memory function, normal aging, and dementia [13,14], presents a higher vulnerability to Aβ_42_ accumulation than the hippocampus in the mouse model of AD Tg2576 [10].

**Figure 2:**
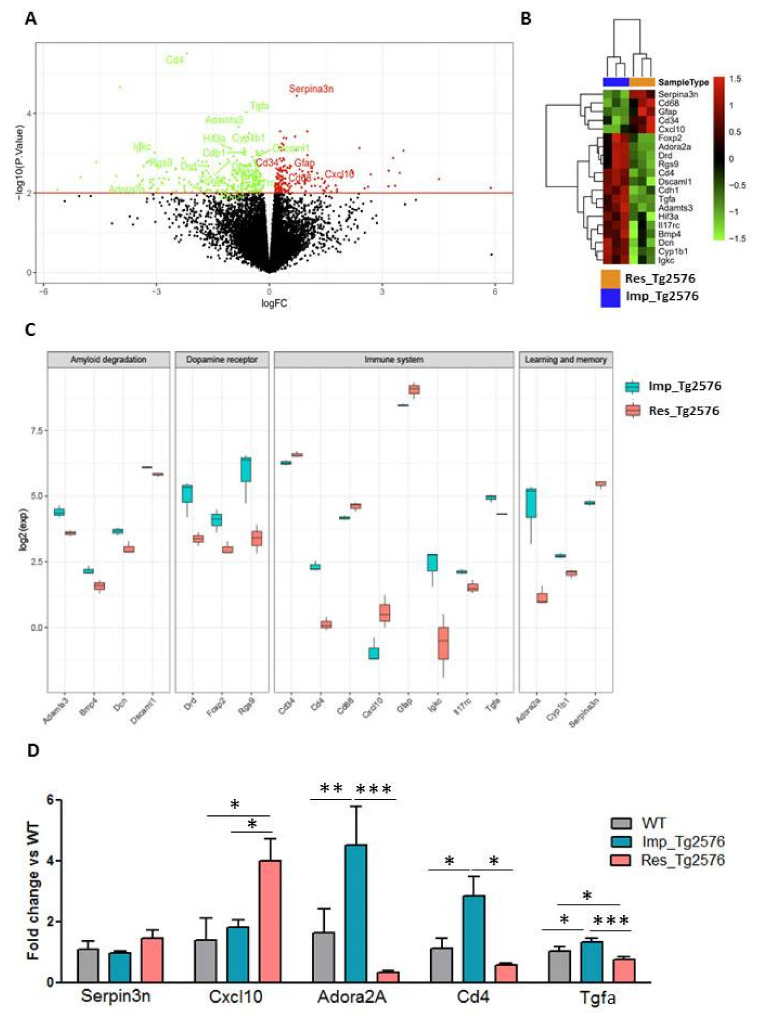
Genes and pathways dysregulated in the prefrontal cortex of cognitive resilient Tg2576 mice by RNA seq. (**A**) Genes and pathways dysregulated in the prefrontal cortex of cognitive resilient Tg2576 mice by RNA seq. (A) Volcano plot of expressed genes in resilient and impaired Tg2576 mice. Fold change between both groups of samples (x axis) is plotted against statistical significance (y axis) for each gene. Genes upregulated with *p* < 0.01 are depicted in red, and those downregulated with *p* < 0.01 are shown in green. (**B**) Heat map illustrating the most significant differentially expressed genes between resilient and impaired mice. Red represents genes with positive log fold-change (log FC), indicating higher expression in resilient compared to impaired mice. Green indicates negative log FC and thus lower expression in cognitive resilient vs. demented mice. (**C**) Box plot of log2 values of the expression of selected transcripts across resilient and impaired Tg2576 mice. (**D**) Prefrontal-cortex mRNA levels of certain selected markers involved in inflammation, learning, and memory that were detected by qRT-PCR in WT, Imp_Tg2576 and Res_Tg2576 mice (one way ANOVA test followed by Newman-Kewls post hoc test, *n* = 3–4, * *p* ≤ 0.05, ** *p* ≤ 0.01, *** *p* ≤ 0.001).

In order to identify the probe sets that showed significant differential expression between the two groups of transgenic animals, a LIMMA analysis [15] was used. Genes were selected using a *p* < 0.01 threshold of significance. These results are publicly available via GEO (under accession number GSE180968). A total of 431 differentially expressed genes were identified. Among them, 164 genes were up-regulated (in red), and 267 genes were down-regulated (green) (Figure 2A volcano plot). Gene ontology (GO) analysis provided further insights into the potential biological processes underlying cognitive resilience (Supplementary Figure S1).

Next, we performed functional analyses of the differentially expressed genes using IPA. Table 1 summarizes the top five canonical pathways; and include communication between innate and adaptive immune cells, CREB signaling in neurons, hematopoiesis from pluripotent stem cells, the neuroprotective role of THOP1 in Alzheimer’s disease, and primary immunodeficiency signaling. The *p* value and the list of the specific genes differentially expressed for each pathway are also depicted in the table.

For further downstream analysis, we selected a list of genes of interest (Supplementary Table S1) that are visualized in a heatmap in Figure 2B. Among these genes, *Serpin3n*, *Cd68*, Gfap, *Cd34,* and *Cxcl10* were found among the genes up-regulated in Res_Tg2576 compared to Imp_Tg2576 mice. However, genes such as *Foxp2*, *Adora2a*, *Drd*, *Rgs9*, *CD4*, *Dscaml1*, *Cdh1*, *Tgfα*, *Adamts3*, *Hif3a*, *Il17rc*, *Bmp4*, *Dcn*, *Cyp1b1,* and *Igkc* were found down-regulated in cognitive resilient compared to impaired Tg2576 mice. As depicted in the box plot in the Figure 2C, an important subset of these genes (*Cd34*, *CD4*, *Cd68*, *Cxcl10*, *Gfap*, *Igkc*, *Il17rc* and *Tgfa*) is related with inflammation. This result is in line with the hypothesis that alterations in neuroinflammation underlie cognitive resilience to AD in humans [16]. A second subset of genes (*Adamts3*, *Bmp4*, *Dcn,* and *Dscaml1)* is involved in amyloid degradation, the accumulation of this peptide being one of the main pathophysiological markers of AD [17]. A third one (*Drd*, *Foxp2 and Rgs9)* is related to dopamine-receptor signaling. It is well-known that alterations in dopamine neurotransmission contribute to cognitive dysfunction [18]. In the same line, a fourth subset of these genes (*Adora2a*, *Cyp1b1* and *Serpin3n*) is implicated in learning and memory, two of the cognitive processes altered in AD [17].

In order to confirm that some of the altered genes are underlying the lack of dementia in cognitive resilient Tg2576 mice, we validated by quantitative real-time PCR (qRT-PCR) in WT, Imp_Tg2576 and Res_Tg2576 mice some of the genes that were found dysregulated in the RNAseq and related with inflammation and learning and memory. As mentioned before, *Serpin3n* and *Cxcl10,* which have been previously linked with AD [19,20], were among the genes differentially upregulated in Res_Tg2576 mice. Although we have shown non-differences in *Serpin3n* mRNA expression between WT, impaired and resilient Tg2576 mice (one way ANOVA with group as variable: F(2,8) = 1.099, *p* = 0.3788), we could confirm that *Cxcl10* mRNA expression was significantly increased in cognitive resilient individuals compared to WT and demented ones (one way ANOVA with group as variable: F(2,8) = 5.233, *p* = 0.0352). Regarding inflammation, as depicted in Figure 2A, the RNAseq showed that *CD4* was the gene most differentially down-regulated in cognitive resilient compared to cognitive impaired mice. Interestingly, we have observed that *CD4* mRNA expression is significantly increased in Imp_Tg2576 mice compared to WT and, indeed, this increase was absent in Res_Tg2576 mice (one way ANOVA with group as variable: F(2,8) = 6.714, *p* = 0.0194). This gene encodes the CD4 membrane glycoprotein of T lymphocytes; an increase in the expression levels of CD4 may indicate an increase in the infiltration of peripheral immune cells into the CNS [21]. The gene *Adora2a* also appeared down-regulated in resilient mice in the RNAseq. This gene encodes for the adenosine A2A receptor (A2AR), which is believed to affect different neuropsychiatric functions by modulating several neurotransmitter systems, including dopaminergic and glutamatergic neurotransmission [22]. Here, we could confirm by qRT-PCR that the expression of this gene was significantly reduced in cognitive resilient mice compared to demented ones (one way ANOVA with group as variable: F(2,19) = 11.51, *p* = 0.0005). Moreover, we showed that *Adora2a* mRNA expression tends to be increased in Tg2576 demented mice compared to their negative littermates. Similar changes were evidenced when analyzing *Tgfa* mRNA expression (one way ANOVA with group as variable: F(2,19) = 11.14, *p* = 0.0006), which is important in controlling proliferation of glial and Schwann cells and the survival of differentiated neurons [23].

### 2.3. Expression Levels of CD4 Transcript in Human AD Samples

Since *CD4* seems to be the most differentially down-regulated gene in the PFC of resilient mice among the inflammatory validated genes and, taking into account its previous association with AD progression [24], further analysis in human AD samples were achieved to confirm its contribution to AD pathophysiology. Brain samples from AD patients with a late Braak stage (IV–VI) and their respective controls (Supplementary Table S2) as well as PBMCs from a cohort of AD and MCI individuals and their age-matched controls (Table 2) were used to determine *CD4* mRNA levels and its association with cognitive status.

**Figure 3:**
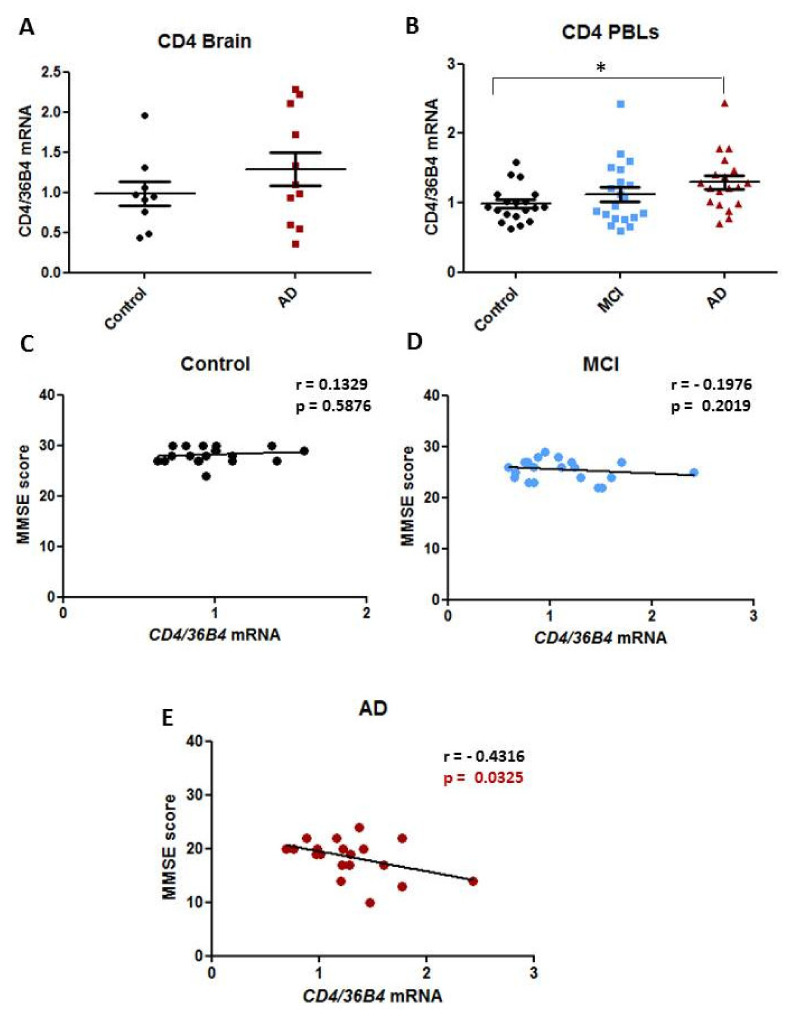
Expression levels of *CD4* mRNA in human AD samples. (**A**) Temporal cortex *CD4* mRNA levels detected by qRT-PCR in samples of AD patients and their respective controls. (**B**) *CD4* mRNA levels detected by qRT-PCR in PBMCs samples from a cohort of AD and MCI individuals and their age-matched controls (* *p* ≤ 0.05; Kruskal-Wallis test, Control vs AD). (**C**–**E**) Correlations between *CD4* mRNA levels and MMSE score in control (**C**), MCI individuals (**D**), and AD patients.

As shown in Figure 3A, the analysis of *CD4* mRNA in brain samples (temporal cortex) revealed no changes between AD and controls, although variability within each group was observed. However, the analysis of *CD4* mRNA in PBMC samples showed a particular immune profile for this transcript in AD samples (Figure 3B); specifically, *CD4* mRNA levels were significantly increased in AD samples when comparing to the control group while in the case of MCI samples, only a tendency to increase was appreciated. Thus, to study the potential functional impact of *CD4* in AD, we correlated *CD4*mRNA levels with MMSE score for each group. Interestingly, a significant linear correlation between *CD4*mRNA at the peripheral level and MMSE score was observed in AD patients (Spearman’s correlation, r  =  −0.43, *p* value  =  0.032, confidence interval −0.7407 to 0.02821, R^2^ 0.1863) (Figure 3E) while this correlation was not present in controls (Spearman’s correlation, r  =  0.13, *p* value = 0.58, confidence interval −0.3421 to 0.5538, R^2^ 0.01766) (Figure 3C) or in MCI individuals (Spearman’s correlation, r  =  −0.19, *p* value = 0.2, confidence interval −0.5887 to 0.2685, R^2^ 0.03904) (Figure 3D). To determine whether peripheral mRNA levels could be associated with cognitive status, data from AD and MCI were analyzed together. Similarly, a significant correlation between *CD4* transcript and MMSE score was observed (Spearman’s correlation, r  =  −0.35, *p* value  =  0.01) (Supplementary Figure S2) suggesting a possible link between peripheral *CD4* mRNA and cognitive decline.

## 3. Discussion

The lack of effective therapies for AD despite years and years of study has highlighted the need to change the way of approaching AD research. Recent evidence has pointed out to the study of resilient individuals as a promising strategy to identify new therapeutic targets for different diseases, including depression and AD [8,25]. Taking into account our previous success on identifying PLA2G4E overexpression as a novel therapeutic strategy for AD by studying cognitive resilient individuals [8], here we performed a similar study to try to better understand the mechanisms underlying dementia resilience in AD.

Once again, we were able to confirm that resilient-aged-Tg2576 individuals can be easily identified by employing the MWM paradigm [8]. This mouse model of amyloidosis, has been extensively used in AD research along the years. Among other AD-neuropathological hallmarks, Tg2576 mice present Aβ accumulation from 7 months and spatial-memory impairments from 12 [26]. As previously reported, Aβ_42_ levels are higher in the prefrontal cortex than in the hippocampus of Tg2576 mice and, taking into account the implication of this area on memory function [10], we used the prefrontal cortex of this model to perform a transcriptomic analysis in order to shed some light into the key players on cognitive resilience. For this purpose, a RNAseq analysis followed by IPA and GO analysis was performed. Taken together, the results obtained pointed out to differences in genes related to neuroinflammation, amyloid degradation, memory function, and neurotransmission as the principal risk factors to develop dementia in the context of AD pathology.

The majority of the genes differentially regulated between resilient and demented Tg2576 mice were related with the immune system. In the last few years, several longitudinal studies performed in asymptomatic or cognitive resilient individuals have suggested that differences in neuroinflammation, specifically astrogliosis and/or microglial activation, could be playing a role in AD cognitive resilience. However, there is some controversy. For example, some studies postulated that an increase in the astrocytic marker GFAP is protective against AD-related dementia [27], which would be in agreement with the increase in *Gfap* mRNA expression observed here in cognitive resilient mice, while others reported the contrary [28]. Apart from the possible involvement of astrocytosis on cognitive resilience, the majority of the results obtained in the present work suggest the contribution of peripheral immune cells infiltration, specifically of CD4 positive T cells, to dementia in AD [29]. FACS analysis would have allowed us to quantify different populations of T cells in the brain, however, the very low level of peripheral cell infiltration prevents this technique from being used in this model. In fact, it is well known that, in AD and related dementias, the involvement of blood-derived immune cells in CNS tissue is limited and the brain-resident macrophages, microglia, appear to be the primary component of the immune system acting locally in the CNS tissue [30]. The higher *CD4* mRNA expression found in cognitive impaired Tg2576 mice could be a consequence of the increased amount of *Adora2A* transcript observed in these mice. In fact, it has been reported that endothelial adenosine 2a receptor (which is encoded by *Adora2A* gene) activation promoted blood–brain barrier (BBB) breakdown in mice with diet-induced insulin resistance [31], thus, this effect may impinge on the infiltration of peripheral immune cells into the brain. In contrast, these results do not seem a priori to align with the increase observed on Cxcl10 mRNA in cognitive resilient individuals, as this chemokine plays a critical role in controlling the entry of several important leukocyte subsets into the brain and it acts as a chemoattractant for CXCR3+ cells, such as activated CD4^+^ T-helper (Th) 1 cells. However, intriguingly, when it is present at high concentrations CXCL10 can be antagonistic for CCR3 [32].

As it has been mentioned before, a subset of the genes altered in cognitive resilient individuals are related with amyloid degradation. Although considering the results obtained in our previous work [8], we do not expect to find differences in amyloid pathology between cognitive resilient and impaired mice, due to the importance of amyloid pathology on AD and, taking into account that differences in compact senile plaques has been previously associated with cognitive resilience in humans [28], this hypothesis should be further explored in the future.

Not surprisingly, two of the other most important processes altered in cognitive resilient Tg2576 mice are related with memory function and neurotransmission. The adenosine receptor 2a, apart from its role in maintaining BBB integrity, is thought to trigger synaptic dysfunction and thus cognitive decline in aging and AD [33]. Further, there is strong evidence that Adora2A is a regulator of synaptic plasticity and, interestingly, the upregulation of this transcript in ageing and in AD is associated with memory impairment. Interestingly, Adora2A receptor blockade prevents memory impairment in aging and in AD models [33,34]. Accordingly, it is has been recently reported that the serum Adora2A levels are increased in AD patients versus healthy individuals [35]. Taking these considerations into account, we can confidently hypothesize that Adora2A is thought to trigger synaptic dysfunction and thus cognitive decline in aging and AD and consequently, the reduction of *Adora2A* transcript observed in cognitive resilient mice may be contributing to the enhanced memory function of these individuals. Pathway analysis on the prefrontal cortex of cognitive resilient and impaired mice also indicated differences in neurotransmission, including dopaminergic transmission. Alterations in the dopaminergic system are frequently found in patients with AD and they have been linked to both cognitive and non-cognitive symptoms. Indeed, it has been reported that dopamine neuronal loss contributes to memory and reward dysfunction in the Tg2576 murine model of AD [36] so it is highly probable that the alterations found on this system are also playing a part on cognitive resilience.

Finally, considering that immune system-related pathways appeared among the top dysregulated ones in the pathway analysis performed on resilient and impaired Tg2576 mice and considering that *CD4* transcript was the most differentially expressed inflammatory marker encountered, *CD4* mRNA expression was further validated in human AD samples, both in the brain and in PBMCs. Although *CD4* mRNA levels were similar between AD and controls in brain samples, at the peripheral level, a significant negative correlation was observed between *CD4*mRNA levels and MMSE score in AD patients. Although no correlation was observed in controls or MCI individuals, it was maintained when both groups (AD and MCI) were analyzed together (Supplementary Figure S2). These data suggest that a peripheral increase of CD4 seems to play a role in cognitive dysfunction associated to AD pathology. Interestingly, Bonotis et al. revealed that although CD4 cells number had a positive relationship with the MMSE score of AD patients, the expression levels of IL-2, a pro-inflammatory cytokine known to be produced by CD4 cells, were negatively correlated with the MMSE score of these patients [37]. Regarding T-cells count in AD, although several studies aimed to evaluate the immune system in people with dementia found no change at any stage of disease progression for total T cells (CD3), CD4 or CD8 T cells [38,39], according to our results, other reports have demonstrated how disease progression in AD seems to parallel with an increase of CD4^+^ T cells in the advanced stages of the disease [24,40]. Likewise, AD patients have higher amounts of pro-inflammatory circulating cytokines compared to healthy controls, also indicating the induction of a systemic immune response (increased number of activated T cells). In fact, several authors suggest that selectively limiting pro-inflammatory CD4^+^ T cells to AD brain could enhance the disease prognosis and that repurposing drug strategies directed to regulate pro-inflammatory CD4^+^ T cells should be considered as plausible therapies for AD [41]. In this context, although our data support this hypothesis, it should be considered that our study is based on measuring *CD4* mRNA levels without differentiating CD4^+^ T cell subpopulations, thus, further analysis would be required to demonstrate the potential role of CD4 T cells in AD and whether its modulation may prevent the development of dementia in AD individuals.

## 4. Conclusions

The RNASeq analysis performed on the prefrontal cortex of cognitive resilient and impaired Tg2576 mice revealed the activation of the peripheral immune system as one of the possible mediators of cognitive resilience mechanisms. Specifically, the *CD4* transcript, which has been previously shown to promote neuroinflammation and cognitive decline in AD [42,43,44], appeared as the most differentially expressed gene between resilient- and demented mice. The analysis of this transcript in AD samples revealed that *CD4* mRNA levels were significantly increased in PBMCs of AD patients compared to controls. Moreover, it showed a linear correlation with MMSE score, demonstrating that peripheral *CD4* mRNA levels could be associated with cognitive status. These findings open the possibility of using CD4 expression as a biomarker for AD and also support the idea that immunotherapy based on CD4^+^ T cells modulation, could be considered as a plausible treatment for AD. Accordingly, it should be interesting in future studies to check whether peripheral Cd4 mRNA levels correlate with cognitive status in Tg2576 mice (both resilient and impaired), and to test in AD models, therapies aimed to reduce CD4 levels, such as anti-CD4 antibodies.

## 5. Methods

### 5.1. Animals

Transgenic aged female Tg2576 mice (16–18 months old), with an inbred C57BL/6/SJL genetic background, were used to identify cognitive resilient mice. This strain overexpresses hAPP with the familial Swedish AD mutation (K670N/M671L) under the control of the prion promoter. Negative littermates were also used as a control group.

Animals were housed 4–6 per cage with free access to food and water, and were kept in a temperature-controlled environment on a 12 h light-dark cycle. All procedures were performed in accordance with current European and Spanish regulations (2010/63/EU; RD52/2013) and the study was approved by the Ethics Committee of the University of Navarra (protocols 113-18).

### 5.2. Morris Water Maze Test (MWM)

The MWM test, which was used to select cognitive resilient mice, was carried out during day time from 9:00 a.m. to 4:00 p.m. Mice were first trained in the visible platform phase (eight trials per day for three days) using a platform above the water surface and with no visible cues. Mice that were able to perform the task under the same conditions (means below 20 s (s) on the third day) went to the hidden platform phase. In this phase, which consists of five consecutive days of training with four trials per day, mice had to locate a submerged platform (1 cm below the water surface) with the help of some visible cues present on the walls of the pool. In both phases, mice were pseudo-randomly placed in selected locations and each test was terminated when the mouse reached the platform or after 60 s. After each trial, mice remained on the platform for 15 s. On the fifth day, mice underwent a probe trial (retention phase) in which they swam in the pool without the platform for 60 s. All tests were monitored by a camera connected to a SMART-LD (Panlab) program for the subsequent analysis of the escape latencies during the visible and hidden platform phases and the percentage of time spent in each quadrant of the pool during the probe tests. All the experimental procedures were performed blind to groups.

### 5.3. Human Brain Samples

Human brain samples (specifically, temporal cortex samples) came from the Navarra Health Service/Osasunbidea’s Research Biobank. They were obtained from patients who met the criteria for the diagnosis of Alzheimer-like pathology with late Braak stage (IV–VI) and symptoms of dementia (*n* = 9), and from non-demented people with no AD pathology (*n* = 10); (Supplementary Table S2). All procedures were carried out in accordance with the Ethics Committee of the University of Navarra (protocol n. 2018.020) and, for all subjects, informed consent was obtained prior to the removal of brain tissue at the time of death and its subsequent use for research. Brain sections were stored at −80 °C until processing.

### 5.4. Human Blood Samples

Human blood samples and data from patients included in the study were provided by the Biobank of the University of Navarra and were processed following standard operating procedures approved by the Ethical and Scientific Committees (protocol n. 2016.022). The samples used for this study corresponded to a cohort recruited by Clínica Universidad de Navarra, which included 20 healthy subjects (controls), 20 mild cognitive impairment patients (MCI), and 19 AD patients. The diagnosis of MCI and AD were made following the recommendations from the National Institute on Aging-Alzheimer’s Association workgroups [45,46]. The demographic characteristics of these patients are included in Table 2.

### 5.5. Purification of Peripheral Blood Mononuclear Cells (PBMC) from Blood Samples

Lithium-heparinized blood samples (20 mL) were poured over 10 mL of Ficoll in a 50 mL tube. After 20 min centrifugation at 600 g the PBMC halo, located between the plasma and Ficoll phases, was collected in a tube and completed with physiological serum to a final volume of 50 mL for washing purposes. Cells were collected by centrifugation for 7 min at 800 *g* and resuspended in 1 mL of physiological serum. Ten million of the cells were taken for RNA purification with Trizol Reagent (Sigma-Aldrich, St. Louis, MO, USA).

### 5.6. RNASeq

RNA was extracted from PFC obtained from Imp_Tg2576 (*n* = 3) and Res_Tg2576 (*n* = 3) using Trizol Reagent (Sigma-Aldrich) and its integrity was confirmed on Agilent RNA Nano LabChips (Agilent Technologies, Santa Clara, CA, USA). Specifically, 1 μg of the total RNA was used to construct cDNA libraries with the TruSeq Stranded mRNA Kit (Illumina, San Diego, CA, USA). The protocol included polyA-selected RNA extraction, RNA fragmentation, random hexamer primed reverse transcription and 100 nt paired-end sequencing was performed by Macrogen (Seoul, Korea) on an Illumina HiSeq platform (Illumina, San Diego, CA, USA) following the 2 × 100 bp paired-end sequencing protocol. The libraries were quantified using quantitative real-time polymerase chain reaction (qPCR) according to the qPCR Quantification Protocol Guide. An Agilent Technologies 2100 Bioanalyzer was used for the qualification. The sequencing data are available on GEO database under the accession number GSE180968.

RNA sequencing data analysis was performed using the following workflow: (i) the quality of the samples was verified using FastQC software version 0.11.8 (https://www.bioinformatics.babraham.ac.uk/projects/fastqc/, accessed on 5 August 2021); (ii) the alignment of reads to the mouse genome (mm10) was performed using STAR [47]; (iii) gene expression quantification using read counts of exonic gene regions was carried out with featureCounts [48]; (iv) the gene annotation reference was Gencode M18 [49]; and (v) differential expression statistical analysis was performed using R/Bioconductor [50]. First, gene expression data was normalized with edgeR [51] and voom [52]. After quality assessment and outlier detection using R/Bioconductor [52], a filtering process was performed. Genes with read counts lower than 6 in the 100% of the samples of all the studied conditions were considered as not expressed in the experiment under study. LIMMA (Linear Models for Microarray Data) [52] was used to identify the genes with significant differential expression between experimental conditions. Genes were selected as differentially expressed using a *p*-value cut off *p* < 0.01. Further functional and clustering analyses and graphical representations were performed using R/Bioconductor [52] and QIAGEN Ingenuity Pathway Analysis.

### 5.7. Quantitative Real-Time PCR

Total RNA was isolated from the correspondent mice or human tissue using Trizol reagent (Sigma-Aldrich). The RNA was treated with DNase at 37 °C for 30 min and reverse-transcribed into cDNA using SuperScript^®^ III Reverse Transcriptase (Invitrogen, Waltham, MA, USA).

Quantitative real-time PCR was performed to quantified gene expression. Assays were done using Power SYBR Green PCR Master Mix (Applied Biosystems, Walthman, MA, USA) and the corresponding specific primers: *Serpin3n* (Fw: 5′GGGATGATCAAGGAACTGGTCT3′, Rev: 5′CCGCGTAGAACTCAGACTTGAA3′), *Clxcl10* (Fw: 5′CCAAGTGCTGCCGTCATTTTC3′, Rev: 5′GGCTCGCAGGGATGATTTCAA3′), *Adora2A* (Fw: 5′GCCATCCCATTCGCCATCA3′, Rev: 5′GCAATAGCCAAGAGGCTGAAGA3′), murine *CD4* (Fw: 5′AGGTGATGGGACCCTACCTCTC3′, Rev: 5′GGGGCCACCACTTGAACTAC3′), *Tgfα* (Fw: 5′CACTCTGGGTACGTGGGTG3′, Rev: 5′CACAGGTGATAATGAGGACAGC3′, human *CD4* (Fw: 5′CAAGGAGGCAAAGGTCTCGAA3′, Rev: 5′CGGCACCTGACACAGAAGA3′) andthe internal control *36B4* (Fw: 5′AACATCTCCCCCTTCTCCTT3′, Rev: 5′GAAGGCCTTGACCTTTTCAG3′). Real-time was carried out using an ABI Prism 7300 sequence detector (Applied Biosystems, Walthman, MA, USA) and data were analyzed using the Sequence Detection software v.3.0 (Applied Biosystems). Relative gene expression was calculated in reference to the control group using the DDCT method.

### 5.8. Data and Statistical Analyses

Unless otherwise indicated, results are presented as mean ± standard error of the mean (SEM). Statistical analysis of the results was done using version 5.03 of GraphPad PRISM. Unpaired two-tailed Student’s *t*-test was performed when comparing two groups and one-way ANOVA followed by Newman-Keuls Multiple comparison post hoc test when comparing more than two groups. For analyzing the latencies to find the platform on the visible and hidden platform phases of the MWM test, a two-way repeated measures ANOVA test (genotype x trial) followed by the Bonferroni’s post hoc test was used. Spearman rank correlation was used to measure the degree of association between *CD4* mRNA and MMSE score in human samples. One-way ANOVA followed by Kruskal-Wallis test was then employed to check if the correlation ratio varies among the groups. Statistical significance was set at * *p* ≤ 0.05, ** *p* ≤ 0.01 and *** *p* ≤ 0.001.

## Figures and Tables

**Table 1:** Top five IPA enriched canonical pathways of the differentially deregulated genes in the frontal cortex of Tg2576 resilient mice versus Tg2576 impaired mice.

IPA Canonical Pathways	*p*-Value	Genes
Communication between Innate and Adaptive Immune Cells	0.0061	CD4, CD79B, CXCL10, Igha, Ighg2b, TNFRSF13C, Trav3-3
CREB Signaling in Neurons	0.0215	ADORA2A, ADRA2B, C5AR2, CATSPER3, DRD1, DRD2, DRD3, GPR101, GPR149, GPR6, GPR88, HTR1D, NTRK1, OPRK1, PTGDR2, SSTR5, TACR1
Hematopoiesis from Pluripotent Stem Cells	0.0240	CD4, Igha, Ighg2b, Trav3-3
Neuroprotective Role of THOP1in Alzheimer’s Disease	0.0287	KLK10, Prss32, TAC1, TMPRSS11A, TMPRSS15, TPSG1
Primary ImmunodeficiencySignaling	0.0373	CD4, Igha, Ighg2b, TNFRSF13C

**Table 2:** Patient demographic and clinical characteristics related to peripheral blood samples (PBMCs).

	Normal Controls	MCI Individuals	AD Patients
Number of subjects	19	20	19
Age (years mean ± S.D.)	73 ± 10.21	77 ± 8.27	77 ± 8.83
Gender (%, women)	53%	45%	47%
MMSE (points mean ± S.D.)	28.2 ± 1.5	25.45 ± 2.01	18.36 ± 3.56

MCI: Mild Cognitive Impairment; MMSE: Mini-Mental State Examination; Age: *p*-value: 0.0384; Gender: *p*-value: 0.095; MMSE score: *p*-value: 0.026

## Data Availability

Supporting reported results are publicly available via GEO (under accession number GSE180968).

## Supplementary Materials

The following are available online at https://www.mdpi.com/article/10.3390/ijms22179120/s1, Figure S1: Gene ontology enrichment analysis on the set of genes differentially expressed between cognitive resilient and impaired aged-Tg2576 mice, Table S1: Selected list of genes differentially regulated in the frontal cortex of Tg2576 resilient mice versus Tg2576 impaired mice, Table S2: Patient demographic and clinical characteristics related to brain samples, Figure S2: Correlation between CD4 mRNA levels and MMSE score in AD and MCI patients.
