# Supplementary material for: Identifying the Main Functional Pathways Associated with Cognitive Resilience to Alzheimer’s Disease
Source: Int J Mol Sci. 2021 Aug 24;22(17):9120. doi: 10.3390/ijms22179120 (PMC8431458; doi:10.3390/ijms22179120)

Supplementary Materials:

Supplementary Figure S1. Gene ontology enrichment analysis on the set of genes differentially expressed between cognitive resilient and impaired aged-Tg2576 mice.

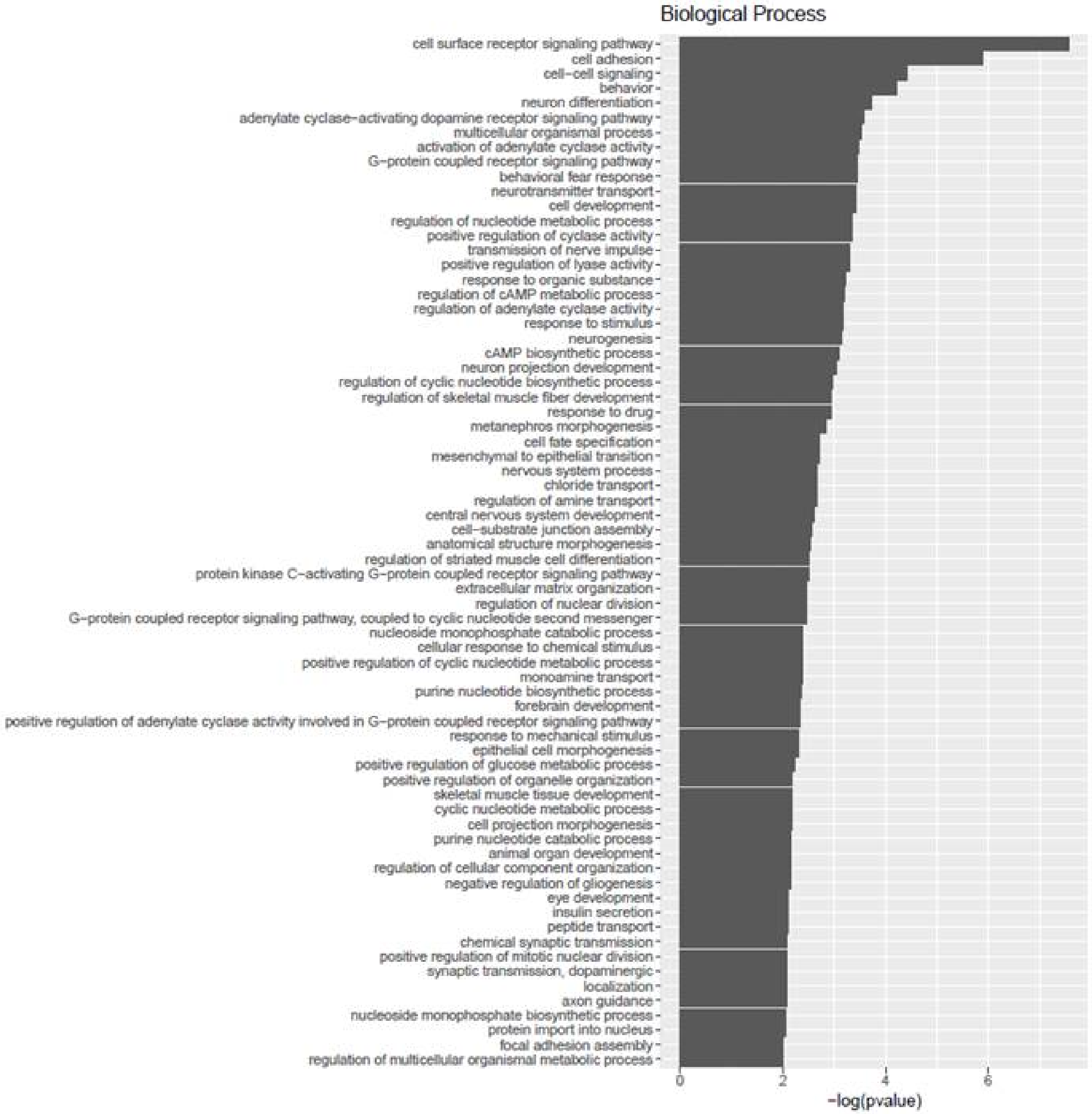

Supplementary Table S1. Selected list of genes differentially regulated in the frontal cortex of Tg2576 resilient mice versus Tg5676 impaired mice.

Gene	logFC	AveExpr	P.Value
Cd4	-2,187747606	1,2263865	3,1300
Tgfα	-0,613002036	4,6245575	9,3700
Serpina3n	0,730005962	5,1015890	3,6300
Adamts3	-0,817526617	4,0028060	0,0002
Hif3a	-1,03325983	3,5322225	0,0007
Cyp1b1	-0,673572171	2,3988862	0,0007
Cdh1	-0,68481697	2,0765840	0,0009
Dcn	-0,660013737	3,3396431	0,0010
Foxp2	-1,073542815	3,5439251	0,0016
Igkc	-3,045181249	0,8722929	0,0009
Drd	-1,626859726	4,1959329	0,0021
Dscaml1	-0,283244744	5,9649883	0,0011
Rgs9	-2,498527091	4,6380467	0,0026
Cd68	0,428387668	4,3997788	0,0026
Gfap	0,572170554	8,7598089	0,0021
Adora2a	-3,395490997	2,8744816	0,0052
Il17rc	-0,579977355	1,8306808	0,0064
Bmp4	-0,601526445	1,8692572	0,0075
clxcl10	1,478093873	0,1663201	0,0051

Supplementary Table S2. Patient demographic and clinical characteristics related to brain samples.

	Normal controls	AD patients
Number of subjects	9	10
Age (years mean ± S.D.)	73 ± 13,64	77,30 ± 7,8
Gender (%, women)	11%	40%
Braak stage	-------------	IV -VI

Supplementary Figure S2. Correlations between CD4 mRNA levels and MMSE score in MCI and AD patients.

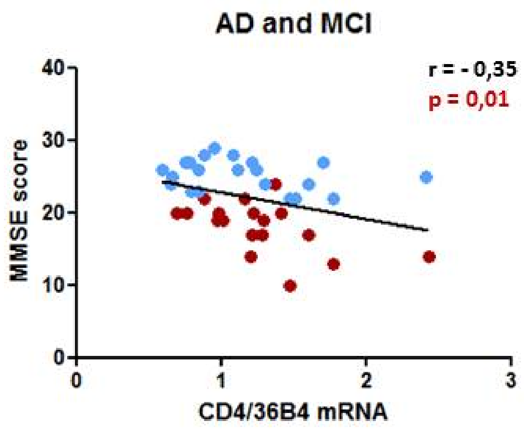

Supplement: Supplementary file 1 [file ijms-22-09120-s001.zip › ijms-1337947-supplementary.pdf]
